# Supplementary material for: Predictive Glycaemic Response of Pasta Enriched with Juice, Puree, and Pomace from Red Cabbage and Spinach
Source: Nutrients. 2022 Oct 31;14(21):4575. doi: 10.3390/nu14214575 (PMC9654938; doi:10.3390/nu14214575)
Supplement: Supplementary file 1 [file nutrients-14-04575-s001.zip › Table S1.pdf]

Table S1. Key technical quality and anti-oxidant capacity of vegetable powder pasta and vegetable puree pasta

Pasta Type	Key Technical Quality				Anti-oxidant Capacity	
	Cooking Loss (g/100g)	Firmness g	Breaking Distance mm	Breaking Force g	Total Phenolic content (mg GAE/100 g)	FRAPS (µmol Fe^{2+} / g)
			Spinach Pasta			
Control	4.40 ± 0.06 [c]	399.75 ± 11.51 [c]	74.28 ± 3.71 [a]	33.55 ± 1.19 [d]	441.21 ± 4.11 [e]	1.26 ± 0.10 [d]
Spinach puree 1%	4.45 ± 0.09 [c]	409.32 ± 11.09 [bc]	71.30 ± 4.88 [a]	41.18 ± 2.23 [b]	490.34 ± 8.18 [c]	2.38 ± 0.32 [bc]
Spinach powder 1%	4.48 ± 0.04 [c]	401.54 ± 9.76 [c]	68.95 ± 4.26 [ab]	37.13 ± 1.95 [c]	473.67 ± 5.95 [d]	2.28 ± 0.30 [c]
Spinach puree 2%	4.80 ± 0.03 [b]	427.46 ± 10.49 [a]	65.69 ± 3.00 [b]	42.34 ± 2.87 [ab]	521.60 ± 2.73 [a]	3.27 ± 0.43 [a]
Spinach powder 2%	4.91 ± 0.05 [a]	419.67 ± 11.25 [ab]	65.21 ± 2.69 [b]	38.12 ± 3.67 [c]	510.66 ± 7.96 [b]	2.84 ± 0.26 [ab]
			Red Cabbage pasta			
Control	4.40 ± 0.06 [c]	399.75 ± 11.51 [a]	74.28 ± 3.71 [a]	33.55 ± 1.19 [a]	441.21 ± 4.11 [e]	1.26 ± 0.10 [e]
Red cabbage puree 1%	4.80 ± 0.07 [b]	388.59 ± 11.96 [ab]	60.90 ± 3.93 [b]	31.41 ± 2.24 [ab]	713.13 ± 9.74 [b]	5.48 ± 0.10 [c]
Red cabbage powder 1%	4.88 ± 0.05 [ab]	396.34 ± 9.95 [a]	59.65 ± 4.21 [b]	31.66 ± 2.62 [ab]	629.24 ± 8.31 [d]	4.45 ± 0.21 [d]
Red cabbage puree 2%	4.94 ± 0.07 [a]	372.80 ± 11.96 [c]	57.45 ± 3.78 [b]	31.12 ± 2.23 [ab]	778.85 ± 7.11 [a]	8.38 ± 0.06 [a]
Red cabbage powder 2%	4.98 ± 0.06 [a]	382.66 ± 8.94 [bc]	57.51 ± 4.04 [b]	30.97 ± 1.79 [b]	679.64 ± 10.15 [c]	6.95 ± 0.12 [b]

Values within a column followed by the same letter are not significantly different from each other ($p > 0.05$) at the same pasta group, according to the ANOVA-Duncan test.
