# Supplementary material for: Predictive Glycaemic Response of Pasta Enriched with Juice, Puree, and Pomace from Red Cabbage and Spinach
Source: Nutrients. 2022 Oct 31;14(21):4575. doi: 10.3390/nu14214575 (PMC9654938; doi:10.3390/nu14214575)
Supplement: Supplementary file 1 [file nutrients-14-04575-s001.zip › Table S2.pdf]

Table S2. HPLC Gradient.

Time (min)	A%	C%
0	68	32
3	68	32
7	30	70
9	30	70
10	68	32
15	68	32

Solvent A, Methanol 100%, Solvent C, Ethyl-acetate 100%
