# Supplementary material for: Predictive Glycaemic Response of Pasta Enriched with Juice, Puree, and Pomace from Red Cabbage and Spinach
Source: Nutrients. 2022 Oct 31;14(21):4575. doi: 10.3390/nu14214575 (PMC9654938; doi:10.3390/nu14214575)
Supplement: Supplementary file 1 [file nutrients-14-04575-s001.zip › Table S3.pdf]

Table S3: Pearson's correlation coefficient (*r*) of Area Under the curve (AUC) of digestive starch versus Fibre and Starch content of vegetable pasta

	**SDF g/100g**	**IDF g/100g**	**TDF g/100g**	**Starch**
		Spinach pasta		
AUC	−0.763 *	−0.751	−0.756 *	0.806 *
		Red cabbage pasta		
AUC	−0.758 *	−0.739	−0.744	0.829 *

*Correlation is significant at 0.05 level; SDF, soluble dietary fibre; IDF, insoluble dietary fibre, TDF; Total dietary fibre
