# Supplementary figures and images for: Predictive Glycaemic Response of Pasta Enriched with Juice, Puree, and Pomace from Red Cabbage and Spinach
Source: Nutrients. 2022 Oct 31;14(21):4575. doi: 10.3390/nu14214575 (PMC9654938; doi:10.3390/nu14214575)

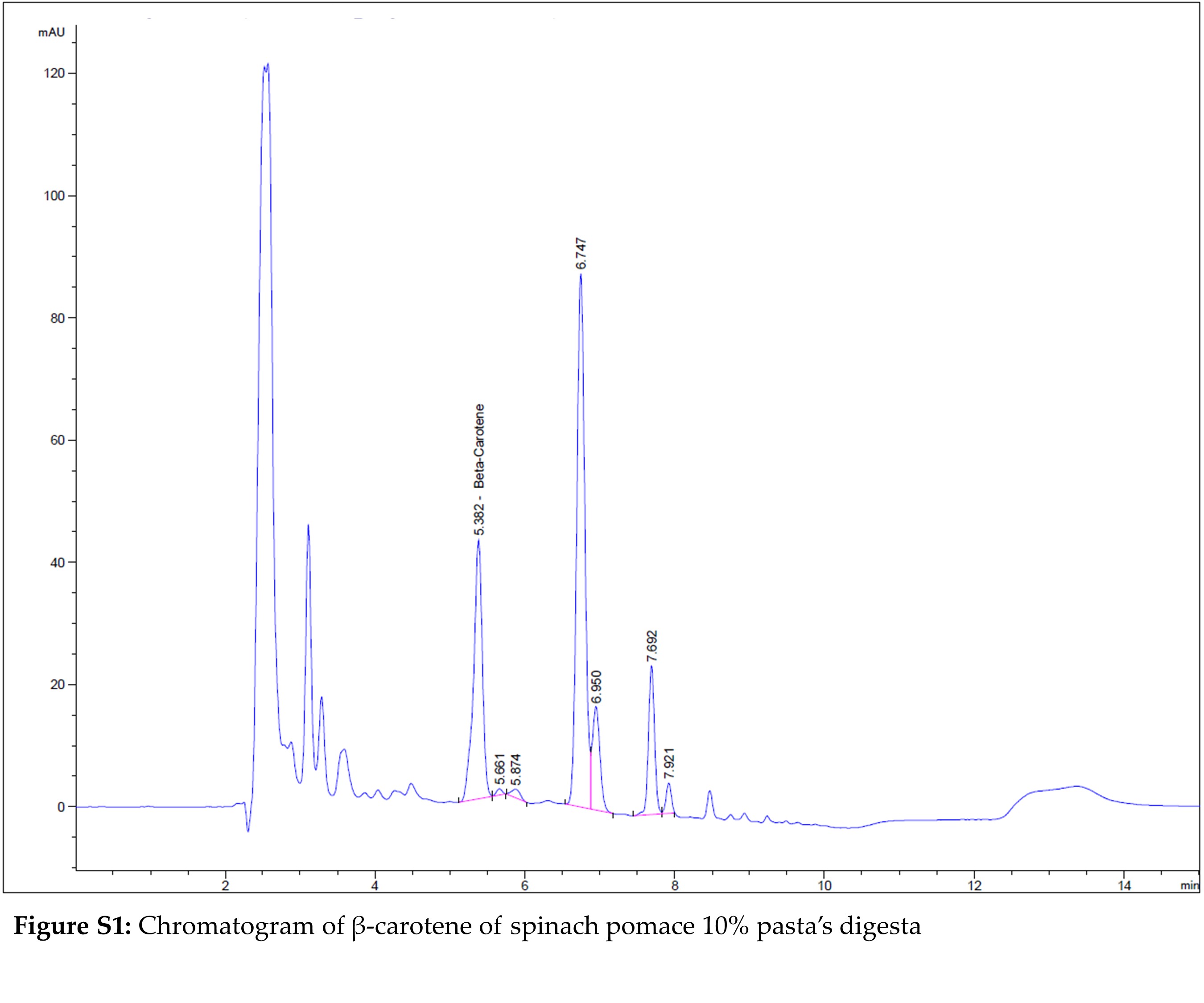

Supplement: Supplementary file 1 [file nutrients-14-04575-s001.zip › Figure S1.jpg]
